# c-CBL/LCK/c-JUN/ETS1/CD28 axis restrains childhood asthma by suppressing Th2 differentiation

**DOI:** 10.1186/s10020-024-00872-1

**Published:** 2024-09-28

**Authors:** Nan Yang, Tianyue Wang

**Affiliations:** Department of Pediatrics, Shengjing Hospital of China Medical University, 36 Sanhao Street, Shenyang, 110004 China

**Keywords:** Asthma, Th2 cells, Inflammation, c-CBL, LCK

## Abstract

**Background:**

Asthma is a common immune disease with high morbidity in children. Type 2 inflammation is the center of asthma development, and mainly mediated by a subset of CD4 + T cells, T helper 2 (Th2) cells. Excess Th2 differentiation was generally associated with asthmatic attack. Casitas B-lineage lymphoma (c-CBL) was reported to involved in T cell development and databank showed its decreased expression in CD4 + T cells from peripheral blood of asthmatic children. This study aims to investigate the role of c-CBL in childhood asthma and Th2 differentiation, and explore the underlying mechanism.

**Methods:**

We collected peripheral blood samples from clinical childhood asthma cases and healthy controls, and determined c-CBL expression in CD4 + T cells. Asthma was induced in neonatal mice by ovalbumin (OVA) intraperitoneal injection and aerosol inhalation, and c-CBL expression in CD4 + T cells from peripheral blood and spleen was measured. Gain-of-function experiments was performed to confirm the effects of c-CBL on Th2 differentiation in vitro. Finally, c-CBL was delivered into asthmatic mice via lentivirus infection to verify its effects on experimental asthma.

**Results:**

c-CBL was lowly expressed in CD4 + T cells from asthmatic children than those of healthy controls. Similarly, it was downregulated in CD4 + T cells from peripheral blood and spleen of asthma mice. Overexpression of c-CBL restrained lung pathological injury and type 2 inflammation in experimental asthmatic mice. Gain-of-function experiments demonstrated that c-CBL inhibited Th2 differentiation of CD4 + T cells from healthy children, and mediated the ubiquitination of lymphocyte cell-specific protein-tyrosine kinase (LCK). LCK acted as a kinase to phosphorylate and activate c-JUN, which was predicted to bind promoter sequence of CD28 by bioinformatic analysis. Dual-luciferase reporter assay verified that c-JUN and ETS1 synergically enhanced transcription of CD28, and this transcription activation was aggravated by LCK overexpression.

**Conclusion:**

c-CBL alleviated asthma and suppressed Th2 differentiation by facilitating LCK ubiquitination, interrupting c-JUN activation and CD28 expression in vivo and in vitro. c-CBL/LCK/c-JUN/ETS1/CD28 axis was partially involved in childhood asthma, and may provide novel insights for clinical treatment for asthma.

**Supplementary Information:**

The online version contains supplementary material available at 10.1186/s10020-024-00872-1.

## Introduction

Asthma is a common chronic, non-communicable disease, and affects 334 million adults and children worldwide (Papi et al. 2018). Asthma usually starts before school age and is responsible for a heavy burden of ill health. Despite availability of effective drugs, a proportion of children with asthma have troublesome symptoms and frequent exacerbations. A toddler clinical study revealed that 26% asthma children did not demonstrate a differential response or have indicators of less disease activity after inhaled corticosteroid/daily leukotriene receptor antagonist/combined treatment with albuterol (Fitzpatrick et al. 2016). Novel treatments or drugs specific to childhood asthma are needed.

An important molecular mechanism of asthma is type 2 inflammation, which occurs in most asthma patients. Type 2 immune response is mainly regulated by a subpopulation of CD4 + T cells known as T helper 2 (Th2) cells. Aeroallergen such as house-dust or pollen stimulates epithelial cells and dendritic cells, and the epithelial tight junctions are cleaved. Activated dendritic cells migrate to lymph nodes, interact with naïve T cells and induce Th2 differentiation (Lambrecht and Hammad 2012, 2015). Mature Th2 cells produce a large number of cytokines, including interleukin (IL)-4, IL-5, IL-6, IL-9 and IL-13, to mediate Th2 inflammation, IgE production and airway injury (Barnes 2001; Robinson 2010). Th2 inflammation has been considered as a target for asthma therapy. So far, omalizumab targeting IgE, mepolizumab targeting IL-5, reslizumab targeting IL-5 receptor and dupilumab targeting IL-4 have been licensed by American Food and Drug Administration (FDA) for treatment of asthma (Normansell et al. 2014; Pavord et al. 2012; Deeks and Brusselle 2017; Bacharier et al. 2021). However, due to the high cost and large dosage of these monoclonal antibodies, novel treatment strategies urgently need to be developed.

Recently, we found the analysis from Gene Expression Omnibus (GEO) databank showed that casitas B-lineage lymphoma (c-CBL) was downregulated in CD4 + cells from childhood asthmatics, comparing with those from heathy children (GSE40887). c-CBL is a RING-type E3 ubiquitin ligase, and involved in immune regulation. c-CBL regulates dendritic cell activation, and its deficiency results in an increase of proinflammatory factors in dendritic cells (Chiou et al. 2011). The dendritic cell-specific ablation of c-CBL leads to spontaneous liver inflammation and fibrosis and early death of the mice (Tong et al. 2021; Xu et al. 2022). c-CBL also regulates T cells, and c-CBL deficiency reverses the neonatal lethality and T cell development interception in SLP-76-/- mice (Chiang et al. 2004). These evidences suggested that c-CBL may be involved in asthma progress via regulating T cell differentiation.

c-CBL belongs to the CBL family, which are well-known negative regulators of receptor tyrosine kinases (RTKs) signaling through their E3 ubiquitin ligase activity (Tang et al. 2022). CBL proteins have a conserved N-terminus composed of a tyrosine kinase binding (TKB) domain, an alpha helical linker region and a catalytic RING finger domain, and facilitate ubiquitination of activated tyrosine kinases (Duan et al. 2004). CBL proteins exert anti-tumor effects by facilitating the degradation of several well-known tyrosine kinases with tumor-promoting roles, such as EGFR and HER2 (Feng et al. 2013; Li et al. 2018).

A previous article reported that c-CBL mediated the ubiquitination and degradation of lymphocyte cell-specific protein-tyrosine kinase (LCK) (Rao et al. 2002). LCK belongs to the Src family of protein tyrosine kinases, and its SH3 domain provides pivotal entry site for TKB domain of CBL proteins (Rao et al. 2002). LCK acts as a regulator of T cell activation, and its inhibitor A-770,041 restrains cockroach extract-induced allergic asthma through attuning Th2/Treg immune response (Alqarni et al. 2022).

Act as a kinase, LCK catalyzed the phosphorylation of transcription factor c-JUN (Shebzukhov et al. 2017). The member of JUN family (c-JUN, JUN-B, JUN-C) generally form a dimer (activator protein-1, AP-1) with FOS or another JUN protein, to binds to DNA sequence to control gene transcription (Shaulian and Karin 2002). Additionally, the crosstalk between AP-1 and another transcription factor ETS1 is found, and they synergistically regulate transcription of some genes, such as *C3AR* and *MMP1* (Schaefer et al. 2005; Pang et al. 2017; Bassuk and Leiden 1995). The analysis of bioinformatic website JASPAR (https://jaspar.elixir.no/) exhibits potential binding sites of both c-JUN and ETS1 in the promoter sequence of *CD28* gene, suggesting their possible regulation on CD28 transcription. CD28 is a T cell-specific surface glycoprotein, and essential for T cell proliferation, survival and cytokine production, and Th2 development. Due to the central role of type 2 inflammation, CD28 is also considered as the therapeutic target of asthma. It has been reported that interruption of CD28-mediated costimulation is highly effective in preventing airway inflammation induce by ovalbumin (OVA) challenge (Gogishvili et al. 2012). It suggested that c-JUN and ETS1 may be involved in regulation of asthma development and Th2 differentiation by controlling CD28 expression, and LCK may function by control c-JUN/ETS1/CD28 axis.

In the present study, we collected peripheral blood from childhood asthma cases and healthy controls, and found that c-CBL was downregulated in the CD4 + T cells from asthmatic children. Subsequently, experimental asthma was induced by OVA challenge in neonatal mice, and c-CBL was also downregulated in CD4 + T cells isolated from peripheral blood and spleen from asthma mice. Gain-of-function experiments were performed in mice and human CD4 + T cells to verify the role of c-CBL in asthma and Th2 development. The regulation of c-CBL on LCK/c-JUN/ETS1/CD28 axis was also investigated.

## Materials and methods

### Clinical sample

The fresh blood samples from twenty cases of childhood allergic asthma and ten healthy childhood volunteers were collected in Shengjing Hospital of China Medical University from Jun. 2022 to Aug. 2022. The ages of childhood asthma cases and healthy volunteers ranged 4–12 years old. Allergic asthma was diagnosed by respiratory physicians. The blood samples from healthy children were collected in conventional health checkup. The sample collection and experiments were conducted in accordance with Declaration of Helsinki, and approved by Ethics Committee of Shengjing Hospital of China Medical University (Approval no. 2023PS1052K).

### Cell isolation and culture

The fresh peripheral blood samples from healthy childhood volunteers were added with equal volume lymphocyte separation medium (human) (Solarbio, Beijing, China), and centrifuged at 1000 g for 30 min at room temperature. The lymphocytes were collected, and were incubated with anti-CD4 microbeads (Miltenyi Biotec Technology & Trading Co., Ltd., Bergisch, Gladbach, Germany) at 4 ℃ for 15 min. The magnetic separation (MS) columns were washed in magnetic field, and the CD4- cells were removed. Subsequently, the CD4 + cells were eluted outside the magnetic field.

The CD4 + cells were restimulated by 50 ng/ml PMA, 2 µM ionomycin for 6 h, and induced Th2 differentiation by incubation with 1 µg/ml anti-CD3, 1 µg/ml anti-CD28, 10 µg/ml anti-IFNγ and 10 ng/ml IL-4 (BioXCell, New Hampshire, USA) for 4 days according to the previous report (Cui et al. 2011).

The coding sequences of human c-CBL (NM_005188.3) and LCK (NM_001042771.1) were inserted into lentivirus vector, which was infected into CD4 + T cells to confirm the role of c-CBL and LCK. Additionally, the coding sequences of human c-CBL and LCK were cloned into pcDNA3.1 vector with flag or HA tag, and the interaction between c-CBL and LCK were verified by transfection of c-CBL-flag and LCK-HA plasmids in 293T cells.

MG132 (5 µM) was used to block proteasome degradation, and cyclohexane (CHX) (25 µg/ml) to intercept translation in CD4 + T cells.

### Animal model

The neonatal C57BL/6 mice were kept in a SPF environment with free access to food and water. In the first part, the mice were derived into two groups: control and asthma (*n* = 6). The asthma mice were intraperitoneally injected with 10 µg ovalbumin (OVA) at postnatal day (P)5 and P10, and then challenged daily from P18 to P20 with aerosolized 1% OVA solution for 10 min (Patel et al. 2017). The control mice were given equal volume of PBS. Twenty-four hours after the last aerosolization challenge, the mice suffered euthanasia. The peripheral blood and spleen was collected for CD4 + T cell isolation, and the lung was used for pathological examination.

In the second part, the mice were derived into four groups: control, asthma, asthma + LV, and asthma + LV-c-CBL (*n* = 12). The lentivirus loaded with mouse c-CBL (NM_007619.1) coding sequence were delivered via intraperitoneal injection two days before the first OVA inhalation of asthma mice. After euthanasia, six mice in every group were used for collecting bronchoalveolar lavage fluid (BALF). Peripheral blood, spleen and lung were collected from other six mice because that bronchoalveolar lavage might induce structure change in lung tissue. Three repeats were performed in each mouse, and six mice in every group were used for every single detection.

The animal care and experiments were in line with Guide for the Care and Use of Laboratory Animals (8th, NIH), and approved by Ethics Committee of Shengjing Hospital of China Medical University (Approval no. 2023PS1035K).

### Real-time PCR

Total RNA was extracted with the TRIpure RNA Extraction Kit (BioTeke Bio., Beijing, China) according to the protocol from manufacturer. After concentration determination, the RNA was reversely transcribed into cDNA using BeyoRT II M-MLV reverse transcriptase (Beyotime Biotechnology, Shanghai, China), with oligo(dT)_15_ and random primer as the primers. The cDNA was applied for real-time PCR to evaluate the expression of human c-CBL, CD28, GATA3 and mouse c-CBL. The PCR procedure was set as follows: 95 ℃ for 5 min 10 s, 60 ℃ for 10 s, 72 ℃ for 15 s, followed with 40 cycles of 72 ℃ for 1 min 30 s, 40 ℃ for 1 min, melting from 60 to 94 ℃ every 1 ℃ for 1 s, and final incubation at 25 ℃ for 1 min. The PCR was performed using Exicycler™ 96 V4 Real-Time Quantitative Thermal Block (Bioneer, Daejeon, Korea). The primers were synthesized by GENERALBIO (Anhui, China), and the sequences were exhibited below:

*homo* c-CBL forward, 5’-GGTACTGAACCCATCGT-3’,

*homo* c-CBL reverse, 5’-AGCACTTGAGGGAACAC-3’;

*homo* CD28 forward, 5’-GGTGCTGGTGGTGGTTG-3’,

*homo* CD28 reverse, 5’-GGTAATGCTTGCGGGTG-3’;

*homo* GATA3 forward, 5’-AAGGCATCCAGACCAGAAA-3’,

*homo* GATA3 reverse, 5’-GCCGGGTTAAACGAGCT-3’;

*mus* c-CBL forward, 5’-CTCCAGTGCCACCAAGA-3’,

*mus* c-CBL reverse, 5’-CTGCTCCAACAGAGTAAGG-3’.

### Western blot

The cellular protein was extracted with RIPA lysis buffer, and the nuclear protein was extracted with the Nuclear and Cytoplasmic Protein Extraction Kit (Beyotime Biotechnology) according to the instruction. After concentration determination, the protein was used for SDS-PAGE (15 µl per lane), and the gel concentration depended on the protein size. After electrophoresis, the protein in the gel was transferred onto PVDF membrane (Thermo Fisher Scientific Inc., Waltham, MA USA), blocked with 5% bovine serum albumin (BSA) for 1 h, and incubated with primary antibody at 4 ℃ overnight. After rinsing with TBST, the protein was incubated with corresponding secondary antibody at 37 ℃ for 45 min, reacted with ECL reagent (ABclonal Technology Co., Ltd., Wuhan, Hubei, China), followed with signal exposure in the dark. The antibody information was shown below: rabbit anti-c-CBL (1:500; Affinity Biosciences, Cincinnati, OH, USA), rabbit anti-LCK (1:500; ProteinTech, Rosemont, Illinois, USA), rabbit anti-c-JUN (1:1000; Affinity Biosciences), rabbit anti-p-c-JUN (Ser73) (1:1000; Affinity Biosciences), rabbit anti-GAPDH (1:10000; Affinity Biosciences), rabbit anti-histone H3 (1:1500; Affinity Biosciences) and goat anti-rabbit IgG-HRP (1:5000; ABclonal Technology Co., Ltd.). GAPDH served as the cellular internal control, and histone H3 as the nuclear internal control.

### Flow cytometry

The cells were firstly treated with 1 µl/ml monensin for 6 h to block the Golgi secretion. Human cells were incubated with human anti-CD3 labeled with PE (LIANKE Biotech., Co., Ltd., Hangzhou, China) and anti-CD4 labeled with FITC (LIANKE Biotech., Co., Ltd.) at 4 ℃ for 30 min in the dark. After permeabilization, the cells were incubated with human anti-IL-5 labeled with APC (Biolegend, San Diego, CA, USA) and anti-IL-13 with PE (Thermo Fisher Scientific Inc.) at 4 ℃ for 30 min in the dark. Then, the percentage of CD3 + CD4 + IL-5 + IL-13 + cells was determined by flow cytometry.

Mouse cells were incubated with mouse anti-CD3 labeled with PE (LIANKE Biotech., Co., Ltd.) and anti-CD4 labeled with APC (LIANKE Biotech., Co., Ltd.) at 4 ℃ for 30 min in the dark. After permeabilization, the cells were incubated with mouse anti-IL-4 labeled with APC (Thermo Fisher Scientific Inc.) at 4 ℃ for 30 min in the dark. The percentage of CD3 + CD4 + IL-4 + cells was determined by flow cytometry.

### Enzyme-linked immunosorbent assay (ELISA)

The OVA-specific IgE content in mouse serum or BALF was assessed with mouse OVA slgE ELISA Kit (FineTest, Wuhan, Hubei, China) according to the manufacturer’s protocols. Briefly, the sample was incubated in the ELISA plate at 37 ℃ for 90 min, and then incubated with HRP-labeled antibody at 37 ℃ for 30 min. Thereafter, the TMB substrate was added into plate to react for 30 min, and terminated with addition of termination buffer. Immediately, the absorbance of the solution was measured at 450 nm with a microplate reader (BioTek, Winooski, VT, USA). The OVA-specific IgE level was calculated based on OD450.

The content of IL-4, IL-5 and IL-13 in human cell supernatant or mouse BALF were evaluated with corresponding ELISA kits (LIANKE Biotech., Co., Ltd.). The details were similar with that of IgE ELISA kit. The sample was incubated in plate at room temperature for 1.5–2 h in vibration, and was incubated with HRP-labeled streptavidin for 30–45 min in vibration. Finally, the plate reacted with TMB substrate for 20 min, and OD450 and OD570 of the solution were assessed.

### Co-immunopercipitation (co-IP)

The protein was extracted from whole cells with Cell Lysis Buffer for Western and IP (Beyotime Biotechnology), and incubated with antibody-linked column at room temperature for 2 h. After washing, the antigen-antibody complex was eluted for western blot to detect HA (1:2000; ABclonal Technology Co., Ltd.), flag (1:4000; ABclonal Technology Co., Ltd.), LCK (1:500; ProteinTech), c-CBL (1:5000; ProteinTech) and c-JUN (1:1000; Affinity Biosciences).

### Dual-luciferase reporter assay

The coding sequence of human EST (NM_001143820.1) or c-JUN (NM_002228.3) was inserted into pcDNA3.1 vector. The promoter sequence of human CD28 (-1655/+58) was inserted into pGL3-basic reporter, and co-transfected in 293T cells with pRT-TK vector, pcDNA3.1-EST, pcDNA3.1-c-JUN and pcDNA3.1-LCK, in presence of Lipofectamine reagent (Invitrogen, Carlsbad, California, USA). Forty-eight hours later, the cells were lysed, and the fluorescence intensity was measured with the Dual Luciferase Reporter Gene Assay Kit (KeyGEN BioTECH, Nanjing, Jiangsu, China), and the luciferase activity was calculated by Firefly/Renilla value.

### Hematoxylin-eosin (HE) staining

One lung lobe was used for pathological examination, including HE, Periodic acid-schiff (PAS) and Masson staining. After immobilization with 10% formaldehyde overnight, the tissue was embedded by paraffin, and cut into 5-µm sections. The sections were deparaffinized with xylene for 15 min twice, and graded ethanol (100% for 5 min twice, 95% for 2 min, 85% for 2 min, 75% for 2 min). Subsequently, the sections were stained with hematoxylin (Solarbio) for 5 min, soaked in 1% hydrochloric acid/ethanol for 3 s, and counterstained with eosin (Sangon Biotech, Shanghai, China) for 3 min. Thereafter, the sections were dehydrated with ethanol and xylene, and sealed with gum. The images were acquired at 200× magnification.

### PAS staining

The paraffin sections were deparaffinized with xylene and graded ethanol, and treated with the Glycogen PAS Staining Kit (Leagene, Beijing, China). Briefly, the sections were oxidated with periodic acid reagent in a humid box for 10 min, interacted with Schiff reagent for 15 min, and counterstained with hematoxylin for 2 min. After dehydration, the sections were sealed with gum. The images were acquired at 200× magnification.

### Masson staining

The paraffin sections were deparaffinized, stained with hematoxylin for 6 min, soaked in 1% hydrochloric acid/ethanol for 3 s, stained with ponceau/fuchsin acid for 1 min. Subsequently, the sections were washed with 0.2% glacial acetic acid, soaked with 1% phosphomolybdic acid for 5 min, and counterstained with aniline blue for 5 min. After washing with 0.2% glacial acetic acid, the sections were dehydrated, and sealed. The images were acquired at 200× magnification.

### Giemsa staining

Giemsa staining was used to detect the cells in BALF. The BALF was dripped onto the glass slide, immobilized with methyl alcohol for 15 min, and treated with Wright-Giemsa Stain Kit (Jiancheng, Nanjing, Jiangsu, China). Briefly, the slides were stained with Giemsa A reagent for 1 min, stained with Giemsa B reagent for 7 min, and washed with water. Then the slides were soaked in 80% ethanol for several seconds, washed, and dried naturally. The numbers of macrophages, lymphocytes, neutrophils and eosinophils were counted under microscope.

### Statistical analysis

The data in this study were presented as mean ± SD in three or six individual experiments. The analysis were performed using GraphPad Prism software. The data from two independent groups were analyzed by unpaired student t test. The data from multiple groups were analyzed by one-way or two-way ANOVA followed with Tukey’s multiple comparisons test. A p value less than 0.05 was considered as statistically significant.

## Results

### c-CBL was decreased in CD4 + T cells from asthmatic children and neonatal mice

**Fig. 1:**
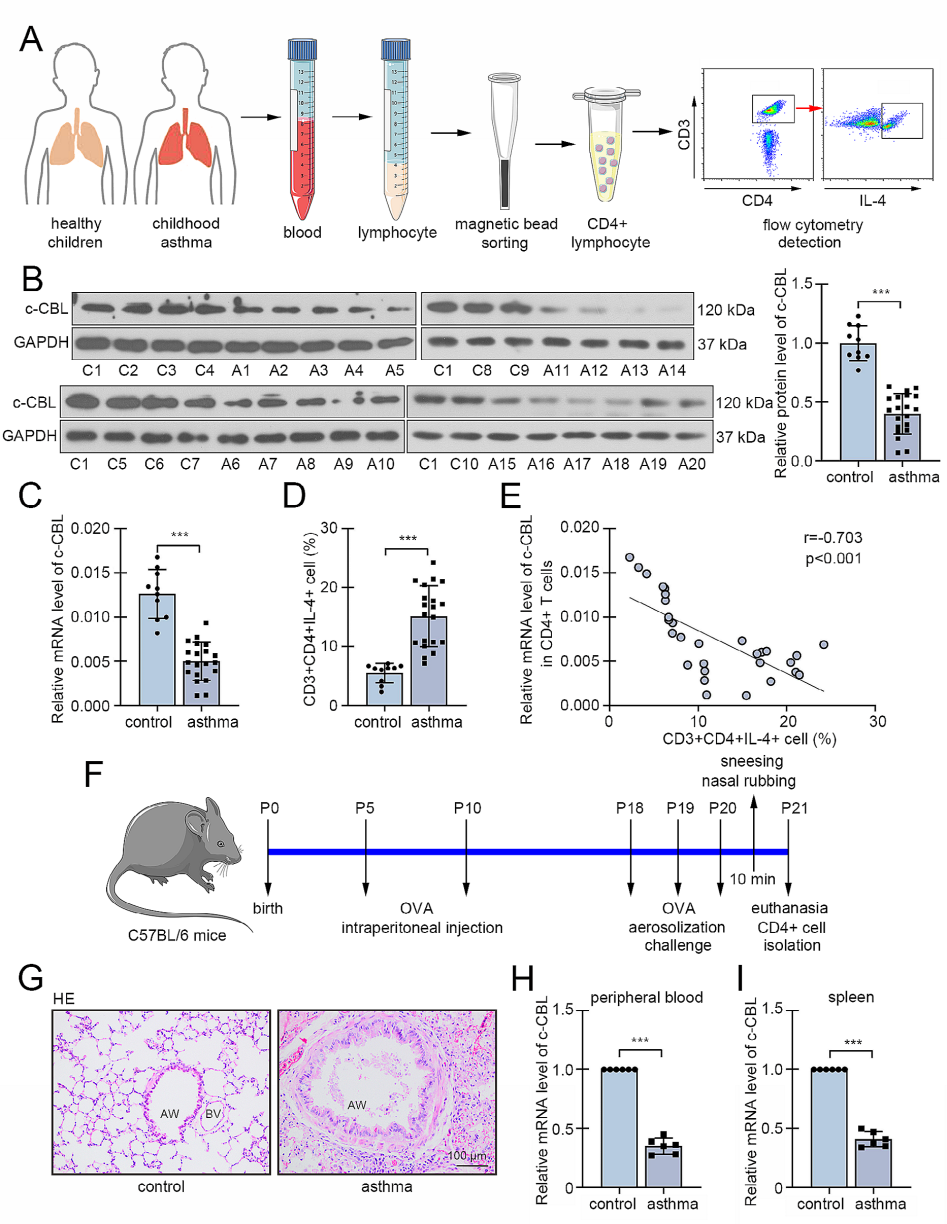
c-CBL was decreased in CD4 + T cells from asthmatic children and neonatal mice. (**A**) Process of CD4 + T cell isolation from childhood asthma cases and healthy children. (**B** and **C**) The expression of c-CBL in CD4 + T cells from blood of asthmatic children and healthy control was determined by western blot and real-time PCR. (**D**) The percentage of Th2 cells in CD4 + T cells. (**E**) The correlation analysis between CD4 + T cell percentage and c-CBL expression in CD4 + T cells from childhood asthma cases and healthy controls. (**F**). The asthma induction and treatment in neonatal mice. G. HE staining of lung tissue from asthmatic and control mice. (**H** and **I**) The mRNA level of c-CBL in CD4 + T cells from peripheral blood or spleen of control and asthma mice. (OVA, ovalbumin; AW, airway; BV, blood vessel; the scale bar represented as 100 μm; **p < 0.01, ***p < 0.001)

The analysis from dataset GSE40887 showed that c-CBL was downregulated in CD4 + T cells from peripheral blood of asthmatic children, comparing with those from healthy controls (Fig. S1). In our study, the peripheral blood was collected from childhood asthma cases and healthy children, and the CD4 + lymphocytes (T helper cells) were isolated by magnetic bead sorting (Fig. 1A). The results from real-time PCR and western blot exhibited that c-CBL was lowly expressed in CD4 + T cells from asthmatic children (Fig. 1B-C). Flow cytometry data revealed the increased percentage of CD3 + CD4 + IL-4 + cells (Th2 cells) in blood of asthmatic children than control (Fig. 1D). The detailed flow cytometry results were shown in Fig. S2. The correlation analysis displayed that the mRNA level of c-CBL in CD4 + T cells was negatively related with the percentage of Th2 cells in blood of asthmatic and control children (Fig. 1E), suggested that the decreased expression of c-CBL may be involved in development of childhood asthma. Subsequently, the asthma was induced in neonatal mice by OVA challenge (Fig. 1F). HE staining showed significant airway dilation, airway wall thickening, and inflammatory cell infiltration around the airway in asthmatic mice (Fig. 1G). Then, the CD4 + T cells were isolated from peripheral blood and spleen in asthma and control mice. The results revealed that the c-CBL expression was significantly reduced in CD4 + T cells from both peripheral blood and spleen of asthma mice (Fig. 1H-I), which was consistent with results from asthmatic children.

### c-CBL facilitated Th2 differentiation of CD4 + T cells

**Fig. 2:**
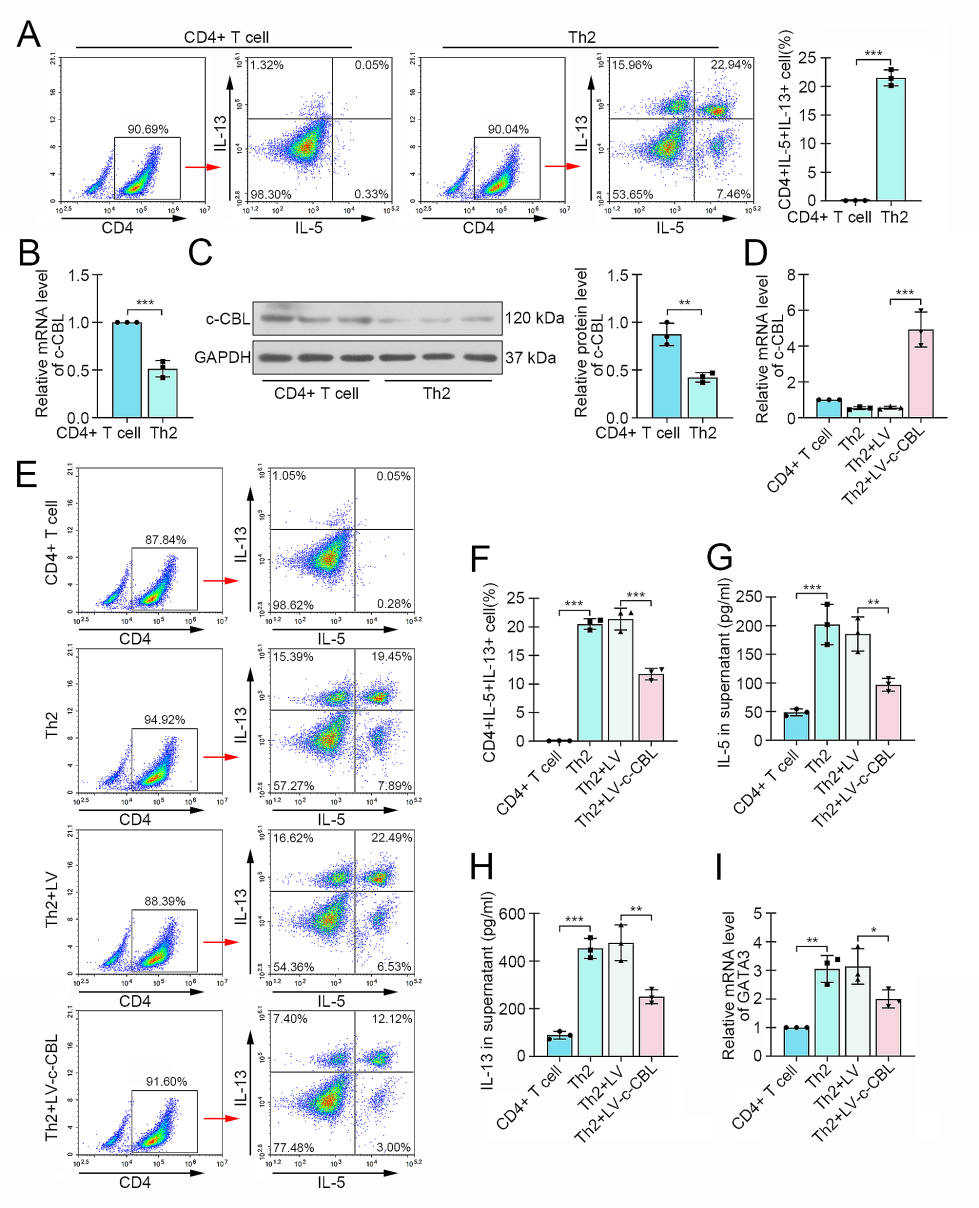
c-CBL facilitated Th2 differentiation of CD4 + T cells. (**A**) The CD4 + T cells were induced to Th2 differentiation, and the percentage of Th2 cells was determined by flow cytometry. (**B** and **C**) The expression of c-CBL in CD4 + T cells with or without Th2 induction was assessed by real-time PCR and western blot. (**D**) c-CBL was overexpressed in CD4 + T cells by lentivirus infection, and the efficiency was confirmed. (**E** and **F**) The Th2 cell percentage in CD4 + T cells with Th2 differentiation induction or/and c-CBL overexpression was evaluated by flow cytometry. (**G** and **H**) The contents of Th2 markers IL-5 and IL-13 in supernatant were detected by ELISA. (**I)**. The expression of Th2 driving molecule GATA3. (*p < 0.05, **p < 0.01, ***p < 0.001)

Thereafter, the CD4 + T cells from healthy children were induced to Th2 differentiation in vitro, and the flow cytometry assay confirmed increased percentage of CD4 + IL-5 + IL-13 + cells (Th2 cells) (Fig. 2A). At the same time, the c-CBL expression in Th2 cells was decreased about 50% after Th2 differentiation in both mRNA and protein levels (Fig. 2B-C). To investigate the role of c-CBL in Th2 differentiation, the lentivirus loaded with c-CBL coding sequence was delivered into CD4 + T cells before Th2 induction. The c-CBL expression efficiency was confirmed by real-time PCR (Fig. 2D). The results from flow cytometry showed that c-CBL overexpression interrupted the Th2 differentiation (Fig. 2E-F). ELISA results displayed reduced production of IL-5 and IL-13 in c-CBL-overexpressed CD4 + T cells (Fig. 2G-H). Consistently, the expression of Th2-specific transcription factor GATA3 was also decreased after enhance expression of c-CBL (Fig. 2I).

### c-CBL bound to LCK and mediated its ubiquitination and degradation

**Fig. 3:**
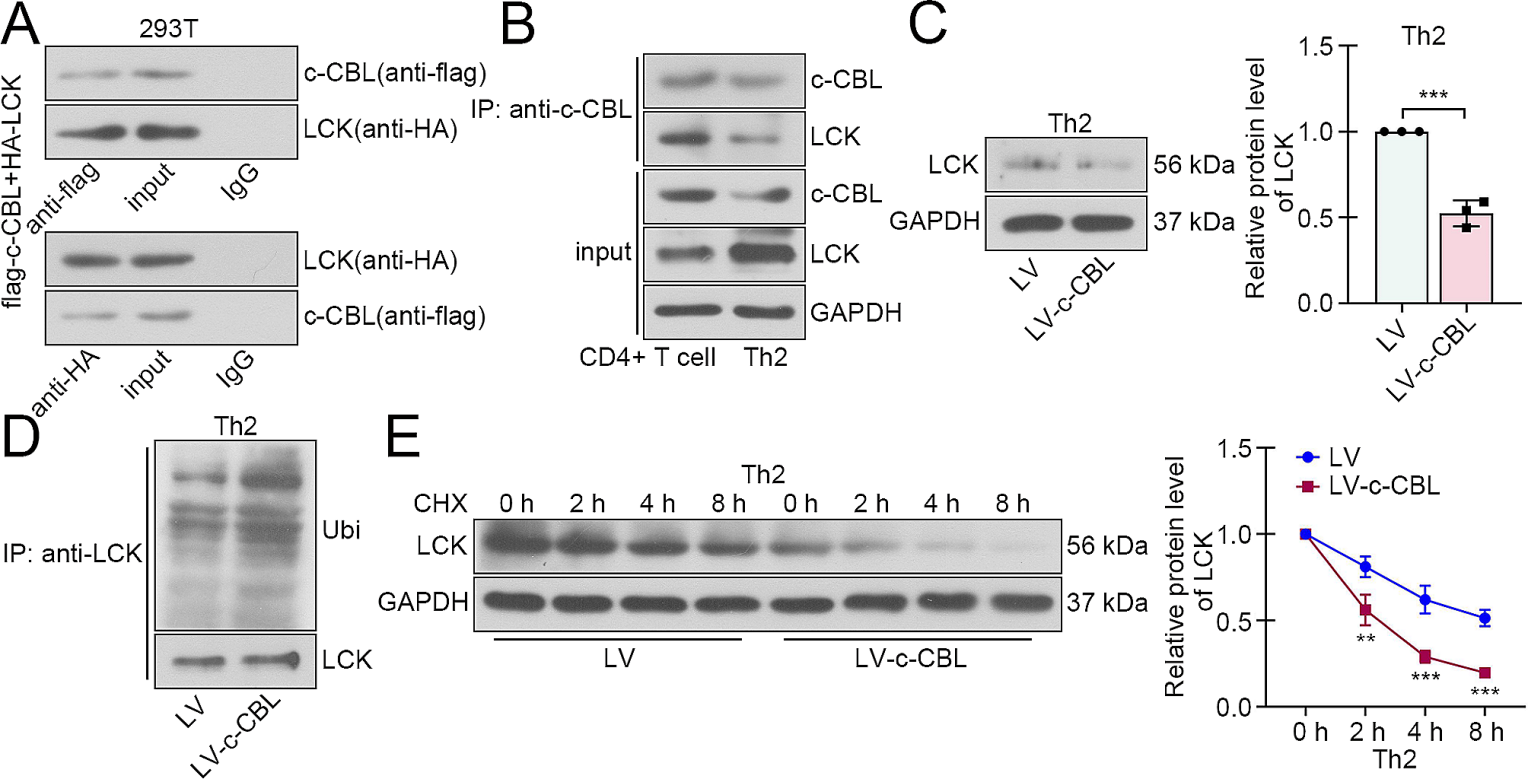
c-CBL bound to LCK and mediated its ubiquitination and degradation. (**A**) The binding between c-CBL and LCK was confirmed by co-IP in 293T cells. (**B**) The interaction between c-CBL and LCK was measured in CD4 + T cells with or without Th2 differentiation induction. (**C**) The LCK expression in Th2 cells after c-CBL overexpression. (**D**) The ubiquitination of LCK was assessed in Th2 cells after c-CBL overexpression. (**E**) The protein level of LCK in c-CBL-overexpressed Th2 cells with CHX treatment for different times. (CHX, cyclohexane; **p < 0.01, ***p < 0.001)

Subsequently, the interaction between c-CBL and LCK was verified in 293T cells by transfection of tag-labeled vectors (Fig. 3A). After Th2 differentiation induction, the expression of LCK was increased, but its interaction with c-CBL was attenuated in CD4 + T cells (Fig. 3B). Then the regulation of c-CBL on LCK was determined in Th2 cells. Western blot results showed that LCK expression was decreased after c-CBL overexpression (Fig. 3C), and IP results displayed that c-CBL promoted the ubiquitination of LCK (Fig. 3D). Ater translation blockage with CHX, the degradation of LCK was accelerated in c-CBL-overexpressed cells (Fig. 3E).

### LCK phosphorylated c-JUN and enhanced its transcription activation on CD28

**Fig. 4:**
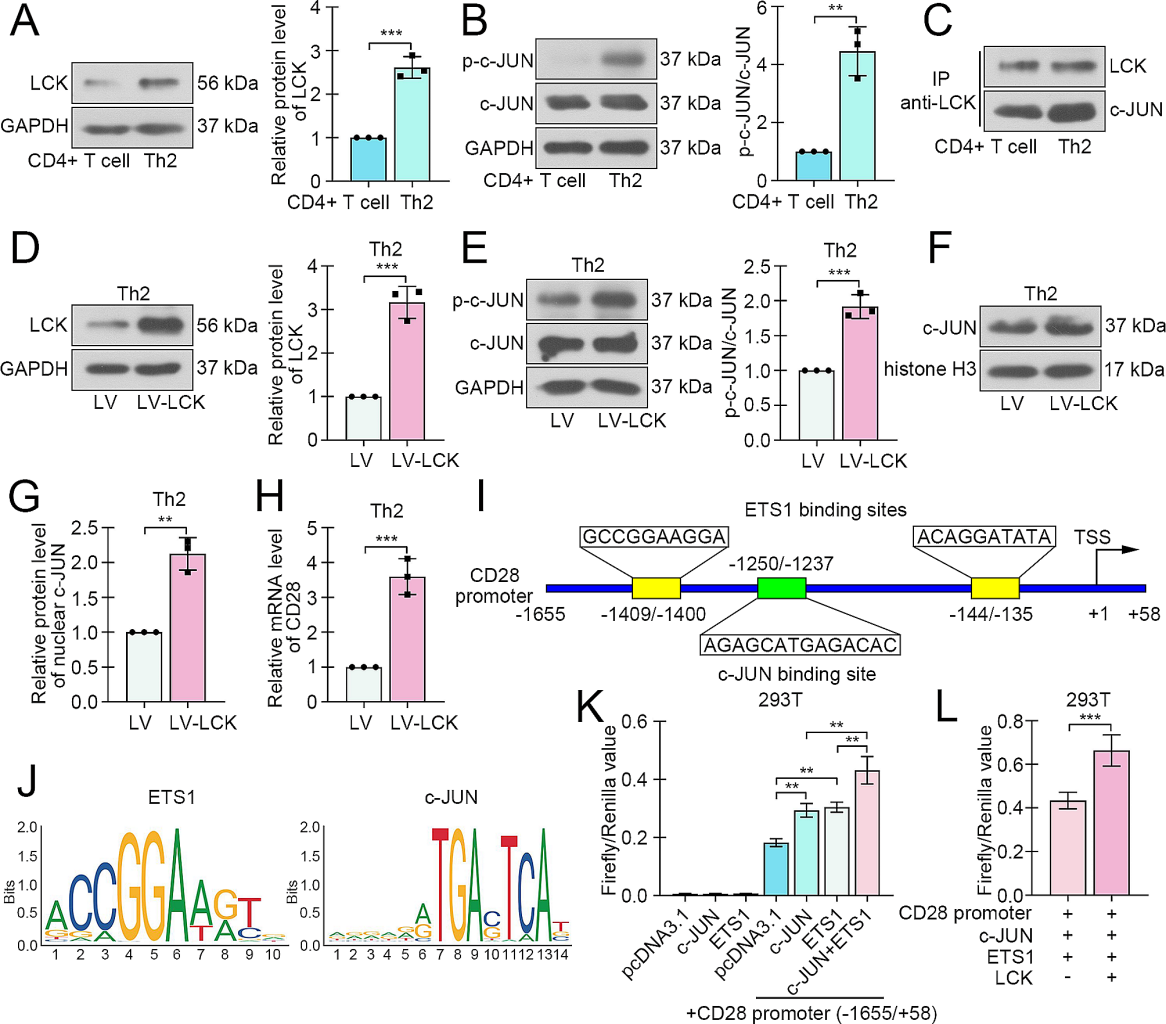
LCK phosphorylated c-JUN and enhanced its transcription activation on CD28. (**A**) The expression of LCK in CD4 + T cells with or without Th2 differentiation induction. (**B)** The expression and phosphorylation (Ser73) of c-JUN in CD4 + T cells with or without Th2 differentiation. (**C**) The binding of LCK and c-JUN in CD4 + T cells. (**D**) The expression efficiency of LCK-overexpressed lentivirus in Th2 cells. (**E**) The expression and phosphorylation (Ser73) of c-JUN after LCK overexpression. (**F** and **G**) The nuclear level of c-JUN in Th2 cells after LCK overexpression. (**H**). The expression of CD28 in Th2 cells after LCK overexpression. (**I**) The potential binding sites of ETS1 and c-JUN to CD28 promoter sequence. (**J**) The conservative binding sequence of transcription factors ETS1 and c-JUN. (**K**) Dual-luciferase reporter assay was used to assess the binding of ETS1 and c-JUN to potential sites of CD28 promoter sequence. (**L**) Dual-luciferase reporter assay with or without LCK overexpression. (**p < 0.01, ***p < 0.001)

Next the function details of LCK in CD4 + T cells were detected. After Th2 differentiation induction, the expression of LCK was increased (Fig. 4A), as well as the phosphorylation of c-JUN (Fig. 4B). Consistently, the binding between LCK and c-JUN was aggravated in Th2 cells (Fig. 4C). To investigate the role of LCK, the lentivirus loaded with LCK coding sequence was infected into CD4 + T cells before Th2 induction. The immunoblotting results revealed that the high expression of LCK induced phosphorylation and nuclear translocation of c-JUN in Th2 cells (Fig. 4D-G). At the same time, the mRNA level of CD28 was also elevated after LCK overexpression (Fig. 4H). The analysis from bioinformatic website exhibited the potential binding sites of c-JUN (-1250/1237) and ETS1 (-1409/-1400, -144/-135) in the promoter sequence of CD28 (Fig. 4I). The conserved binding sequences of ETS1 and c-JUN were shown in Fig. 4J. The dual-luciferase reporter assay revealed that both c-JUN and ETS1 bound to CD28 promoter and facilitated its transcription, and the superposition effect of both was more obvious (Fig. 4K). Moreover, the effects of c-JUN and ETS1 on CD28 transcription was further exacerbated by LCK overexpression (Fig. 4L).

### The effect of c-CBL on Th2 differentiation was abolished by LCK overexpression

**Fig. 5:**
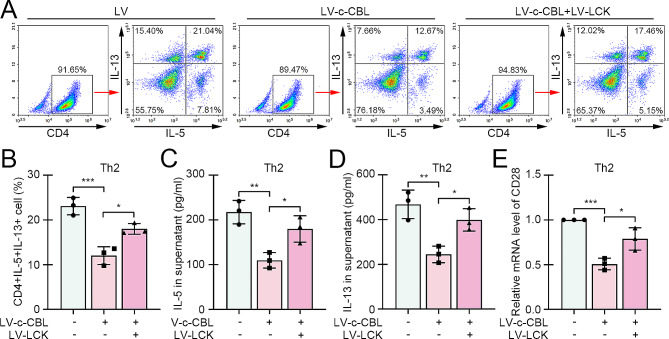
The effect of c-CBL on Th2 differentiation was abolished by LCK overexpression. (**A** and **B**) The Th2 cell percentage after overexpression of c-CBL or/and LCK was determined by flow cytometry. (**C** and **D**) The content of Th2 markers IL-5 and IL-13 in supernatant was measured by ELISA. (**E**) The mRNA level of CD28 after overexpression of c-CBL or/and LCK. (*p < 0.05, **p < 0.01, ***p < 0.001)

To verify the function mechanism of c-CBL in regulating Th2 differentiation, the lentiviruses loaded with c-CBL or LCK coding sequence were simultaneously infected into CD4 + T cells before Th2 induction. Flow cytometry data showed that the Th2 differentiation was delayed by c-CBL, while accelerated by LCK (Fig. 5A-B). Similarly, c-CBL-induced production suppression of Th2 cytokines IL-5 and IL-13 was recovered by LCK overexpression in CD4 + T cells (Fig. 5C-D). Additionally, the expression of CD28 was inhibited by c-CBL and promoted by LCK (Fig. 5E).

### c-CBL alleviated experimental asthma and suppressed Th2 cell activity in neonatal mice

**Fig. 6:**
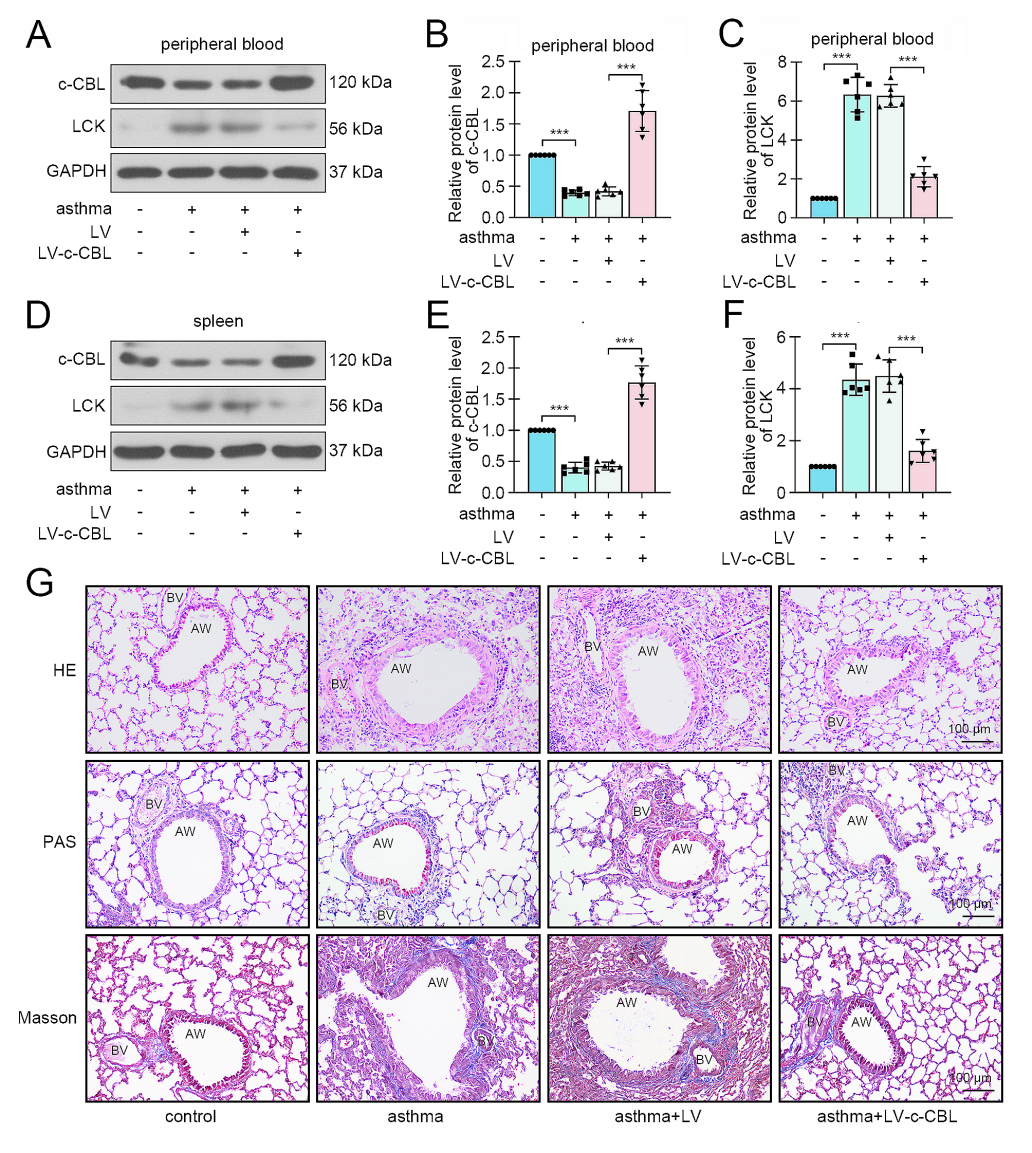
c-CBL alleviated experimental asthma in neonatal mice. The overexpression of c-CBL via lentivirus intraperitoneal injection in experimental childhood asthma mice. (**A**-**C**) The expression of c-CBL and LCK in CD4 + T cells from peripheral blood of mice. (**D-F**) The expression of c-CBL and LCK in CD4 + T cells from spleen of mice. (**G**) HE, PAS and Masson staining were performed to detect the pathological change, glycogen and collagen in lung tissues. (OVA, ovalbumin; AW, airway; BV, blood vessel; HE, hematoxylin-eosin; PAS, Periodic acid-schiff; the scale bar represented as 100 μm; *p < 0.05, **p < 0.01, ***p < 0.001)

**Fig. 7:**
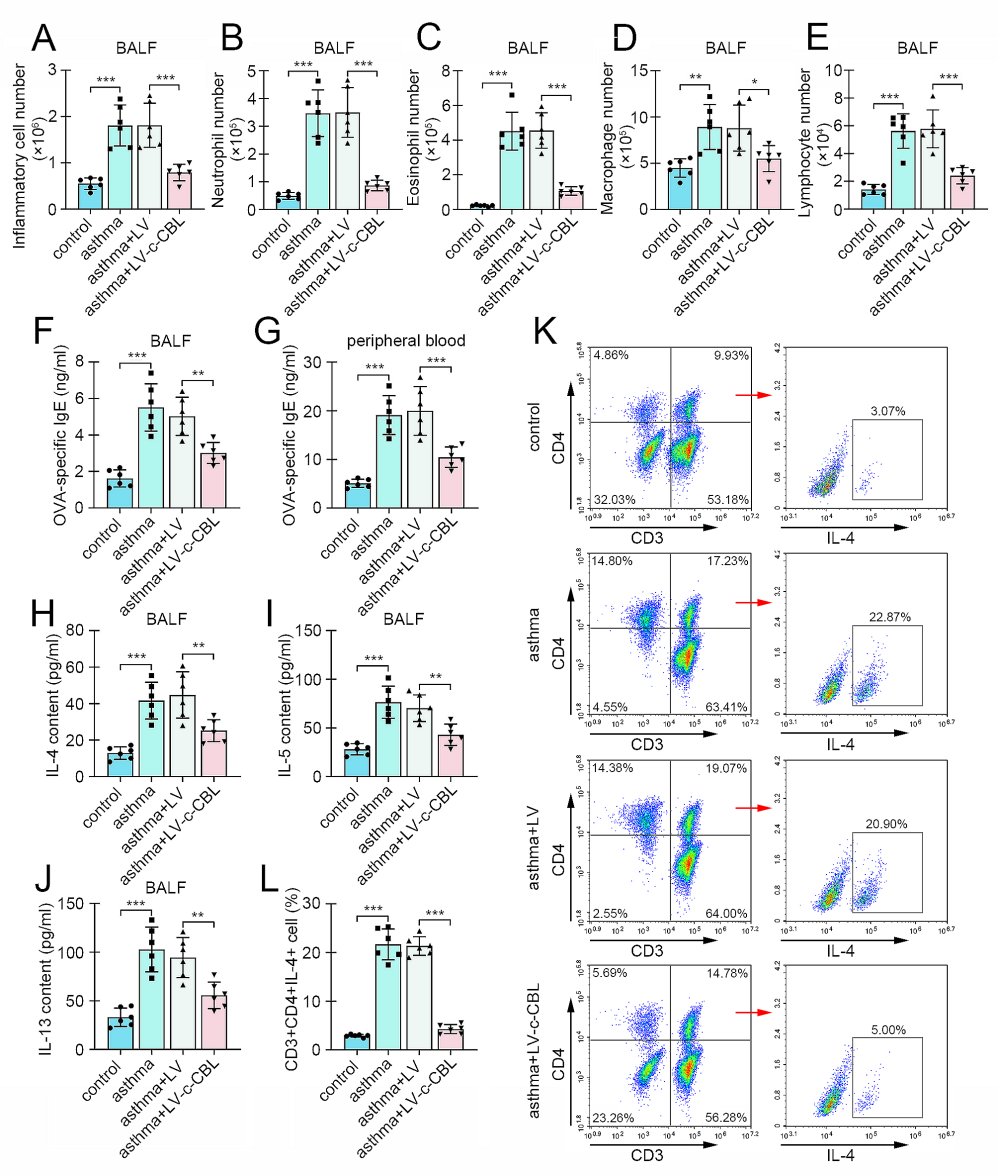
c-CBL suppressed Th2 cell activity in asthmatic mice. (**A-E**) The numbers of inflammatory cells, neutrophils, eosinophils, macrophages and lymphocytes in BALF were counted by Giemsa staining. (**F** and **G**) The OVA-specific IgE levels in BALF and peripheral blood from asthmatic mice with or without c-CBL overexpression were assessed by ELISA. (**H-J**) The contents of Th2 markers IL-4, IL-5 and IL-13 in BALF. (**K** and **L**) The Th2% in lung tissue. (OVA, ovalbumin; BALF, bronchoalveolar lavage fluid; *p < 0.05, **p < 0.01, ***p < 0.001)

In order to explore the effects of c-CBL on asthmatic mice, the c-CBL-overexpressed lentivirus was delivered into mice. The high expression of c-CBL was confirmed in CD4 + T cells from peripheral blood and spleen in asthmatic mice with lentivirus injection. Meanwhile, the expression of LCK was decreased in c-CBL-overexpressed mice (Fig. 6A-F). Pathological staining revealed that the ectopic expression of c-CBL restrained OVA-induced airway injury, inflammatory cell infiltration, mucus production and airway remodeling in neonatal mice (Fig. 6G). Giemsa staining data revealed that the numbers of inflammatory cells, neutrophils, eosinophils, macrophages and lymphocytes were significantly increased in BALF of OVA-challenged mice, and decreased after c-CBL overexpression (Fig. 7A-E). The contents of an anaphylaxis marker OVA-specific IgE in BALF and blood were also elevated in asthma mice, and reduced by c-CBL (Fig. 7F-G). Then the expression of Th2 cytokines was determined in BALF. ELISA results exhibited that the increased IL-4, IL-5 and IL-13 were all suppressed in c-CBL-overexpressing-lentivirus-infected mice (Fig. 7H-J). Finally, the CD4 + CD4 + IL-4 + cells (Th2 cells) were isolated from lung tissue of mice. Flow cytometry showed that the percentage of Th2 cells was significantly increased in OVA-induced asthmatic mice, and decreased after c-CBL deliver (Fig. 7K-L).

## Discussion

**Fig. 8:**
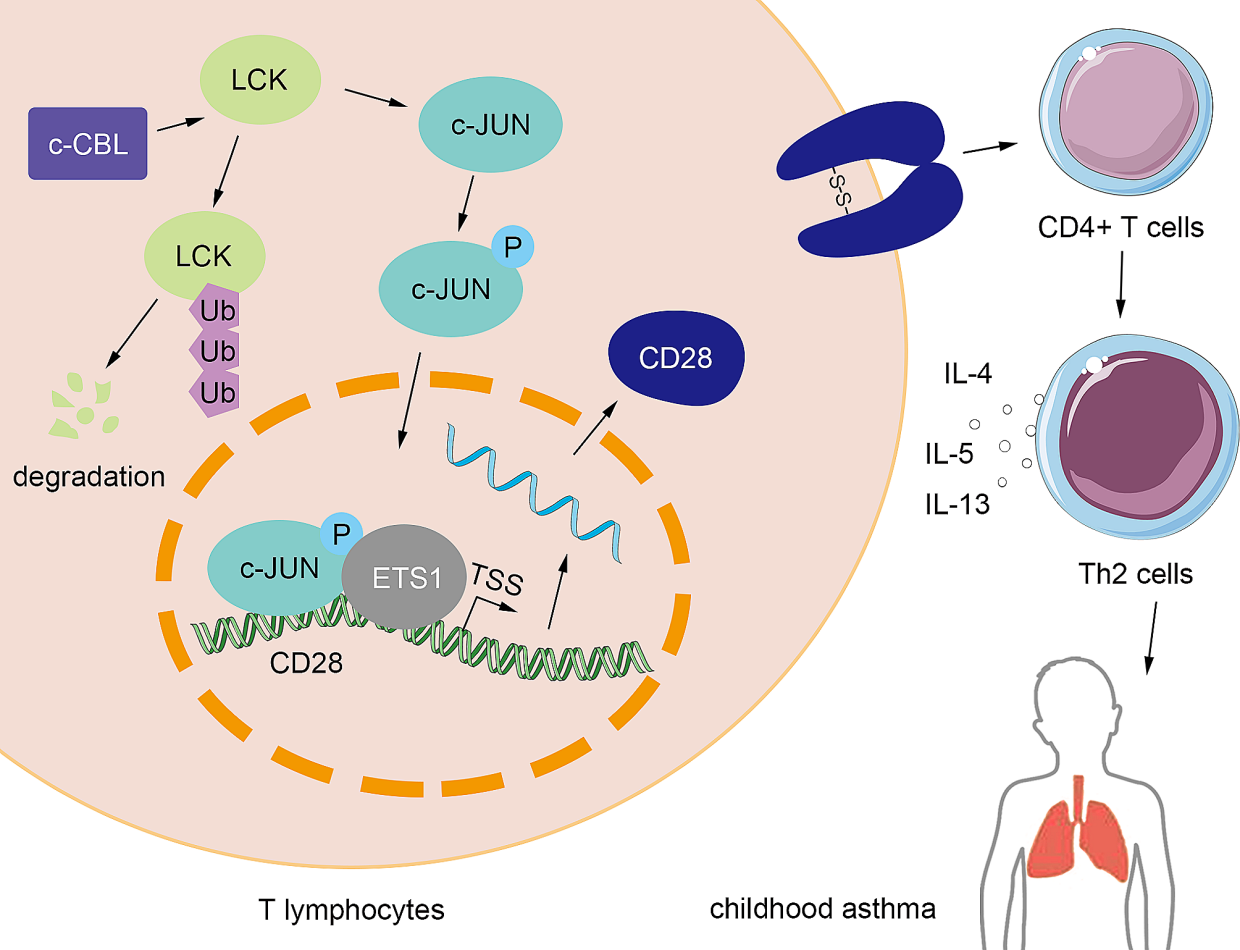
The mechanism of c-CBL/LCK/c-JUN/ETS1/CD28 regulation on childhood asthma. c-CBL catalyzed the ubiquitination of degradation of LCK; LCK mediated the phosphorylation of c-JUN; phosphorylated c-JUN and ETS1 synergistically promoted the transcription of CD28; CD28 homodimers facilitated the Th2 differentiation and asthma development

In the present study, we demonstrated that c-CBL was decreased in CD4 + T cells from blood of asthmatic children and experimental asthmatic neonatal mice. The overexpression of c-CBL restrained OVA-challenged asthma and inhibited Th2 development in mice. In human CD4 + T cells, c-CBL interrupted Th2 differentiation by promoting ubiquitination and degradation of LCK, blocking the phosphorylation of c-JUN, and suppressing its synergetic transcription activation with ETS1 on CD28 (Fig. 8). c-CBL/LCK/c-JUN/ETS1/CD28 axis was beneficial for asthma, and may provide novel targets for asthma therapy.

The CBL family of proteins (c-CBL, CBL-b, CBL-c) in mammal possess conserved domains, and also have similar functions (Lupher et al. 1999). CBL-b was previously reported to restrain asthmatic injury and proallergic Th2 development (Qiao et al. 2014; Carson WFt, Guernsey et al. 2015), which was consistent with our results of c-CBL. Additionally, Chiang et al. demonstrated that CBL-b regulated T cell activation in a CD28-dependent manner (Chiang et al. 2000). CD28 is a T cell-specific molecule, and indispensable for T cell activation. In a large number of papers, including our study, CD28 antibody was used as a ligand in medium to stimulate CD4 + T cells. CD28 enhances production of IL-4 and IL-10 in T cells, and mediates type 2 inflammation (Blotta et al. 1996). Therefore, CD28 is considered as a therapeutic target of asthma, blocking CD28-mediated costimulation is highly effective in preventing OVA-induced airway inflammation (Gogishvili et al. 2012). Our data, together with the results from Chiang, proved that both c-CBL and CBL-b regulated the expression of CD28. However, the regulation details of CBL-b on CD28 was not clear. Our data revealed that c-CBL indirectly regulated CD28 transcription by catalyzing the ubiquitination of LCK and intercepting c-JUN phosphorylation.

CBL proteins were identified by mediating the ubiquitination of tyrosine kinases. It is possible for CBL proteins to catalyze same substrate because of their high similarity. LCK is a non-receptor tyrosine kinase, a suitable substrate of CBL proteins. Our data showed that c-CBL interacted with LCK and mediated its ubiquitination. However, whether CBL-b or CBL-c bind to LCK remains unclear. Inhibition of LCK mitigated airway inflammation and Th2 development in asthma mice (Alqarni et al. 2022, 2023). Our data showed that the binding between c-CBL and LCK was attenuated in Th2 cells, may due to the reduced expression of c-CBL. This suggested that the decreased expression of c-CBL may contributed to LCK function in Th2 differentiation and asthma development.

Our study suggested that LCK phosphorylated c-JUN and promoted its synergistic transcription activation with ETS1 on CD28, and this may be the function mechanism of LCK in Th2 cells. In our previous paper, we found that ETS1 facilitated Th2 differentiation of CD4 + T cells, but it was unclear how it functioned (Wang et al. 2021). The present data suggested ETS1 may play its role by promoting CD28 transcription. Wang et al. reported that ETS1 and AP-1 synergistically promoted the transcription of IL-5 in T cells (Wang et al. 2006). Both CD28 and IL-5 were important for Th2 development and type 2 inflammation, so we speculated that c-JUN and ETS1 may function by regulating multiple genes, not only CD28 and IL-5. Similarly, CD28 may be regulated by other molecules. Therefore, the interaction between c-CBL and LCK, or LCK and c-JUN, may be incomplete. c-CBL/LCK/c-JUN/ETS1/CD28 axis may be regulated by other upstream or downstream molecules, and this need more evidences to elucidate. Anyhow, the effects of c-CBL on childhood asthma was firstly reported, and this may provide novel insights for treatment of asthmatic children.

Asthma is an immune disease characterized by coughing, chest tightness and difficulty breathing. Asthma includes allergic asthma and non-allergic asthma, with the former accounting for the majority of asthma patients, especially children. The type 2 inflammation in allergic asthma is mainly mediated by Th2 cells, along with the elevation of the marker of allergic reaction, IgE, to activate monocytes and macrophages, and increase airway mucus production (Barnes 2001; Robinson 2010). Non-allergic (intrinsic) asthma often develops later in life, has neither IgE reactivity to allergens in the serum nor any obvious involvement of the adaptive immune system such as Th2 cells (Lambrecht and Hammad 2015). In non-allergic asthma, the airway inflammation was mediated by group 2 innate lymphoid cells (ILC2), a class of non T and non B cells that activated by epithelial-derived IL-25 or IL-33 (Kato 2019). Activated ILC2 produce Th2 cytokines, and caused eosinophilia (Yasuda et al. 2012). In our study, the clinical asthmatic children only included allergic asthma due to the uncommon of non-allergic asthma in children. c-CBL was decreased in blood CD4 + T cells from allergic asthma children and animals, and its overexpression ameliorated OVA-challenged allergic asthma in the neonatal mice. However, its expression in non-allergic patients and its effects on non-allergic asthma or ILC2 were still unclear. Due to the high incidence of allergic asthma in children, we focused on childhood allergic asthma and Th2 cell development. Our findings suggested that the increased c-CBL in T cells may contribute to the allergic childhood asthma improvement, although it was still challenging how to achieve the high expression of c-CBL in patients safely and effectively.

In addition, the interaction among c-CBL, LCK, c-JUN, ETS1 and CD28 was confirmed in human cells, and whether this axis is conserved among species needs to demonstrate by more evidences. Moreover, the c-CBL/LCK/c-JUN/EST1/CD28 axis may be a small part of the complex regulation network of allergic childhood asthma, and may control other molecules or be controlled by other molecules. We hope our findings could provide insights for clinical treatment or fundamental investigation of allergic childhood asthma.

## Conclusion

In the present study, we found that c-CBL was lowly expressed in CD4 + T cells from peripheral blood from asthma children and OVA-challenged neonatal mice. Overexpression of c-CBL alleviated airway remodeling and type 2 inflammation in OVA-induced experimental asthmatic mice. c-CBL inhibited Th2 differentiation of CD4 + T cells from healthy children, and promoted ubiquitination and degradation of LCK. c-JUN was phosphorylated and activated by LCK, and synergistically enhanced the transcription of CD28 with ETS1. These findings elucidated the roles of c-CBL/LCK/c-JUN/ETS1/CD28 in asthma and Th2 development, and may provide novel insight therapeutic targets for asthma in clinic.

## Electronic supplementary material

Below is the link to the electronic supplementary material.

Supplementary Material 1

Supplementary Material 2

## Data Availability

Data will be made available on request.
