# Supplementary figures and images for: c-CBL/LCK/c-JUN/ETS1/CD28 axis restrains childhood asthma by suppressing Th2 differentiation
Source: Mol Med. 2024 Sep 28;30:164. doi: 10.1186/s10020-024-00872-1 (PMC11439220; doi:10.1186/s10020-024-00872-1)

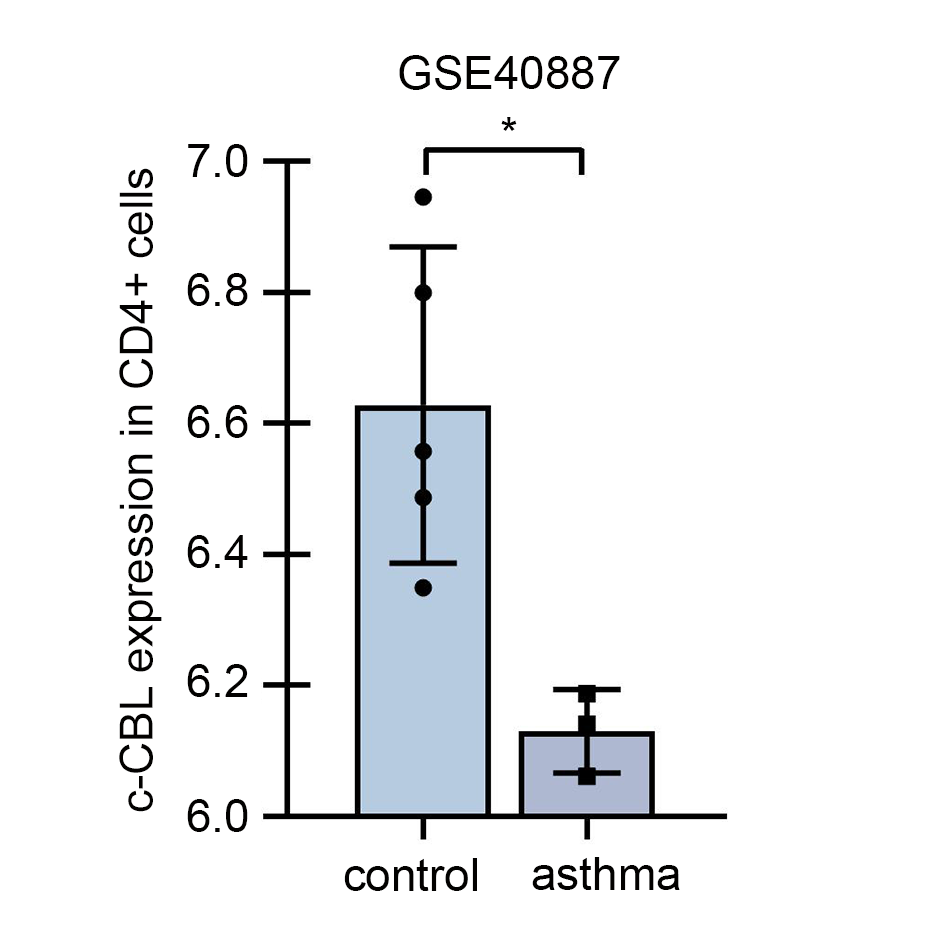

Supplement: Supplementary file 1 — Supplementary Material 1 [file 10020_2024_872_MOESM1_ESM.png]

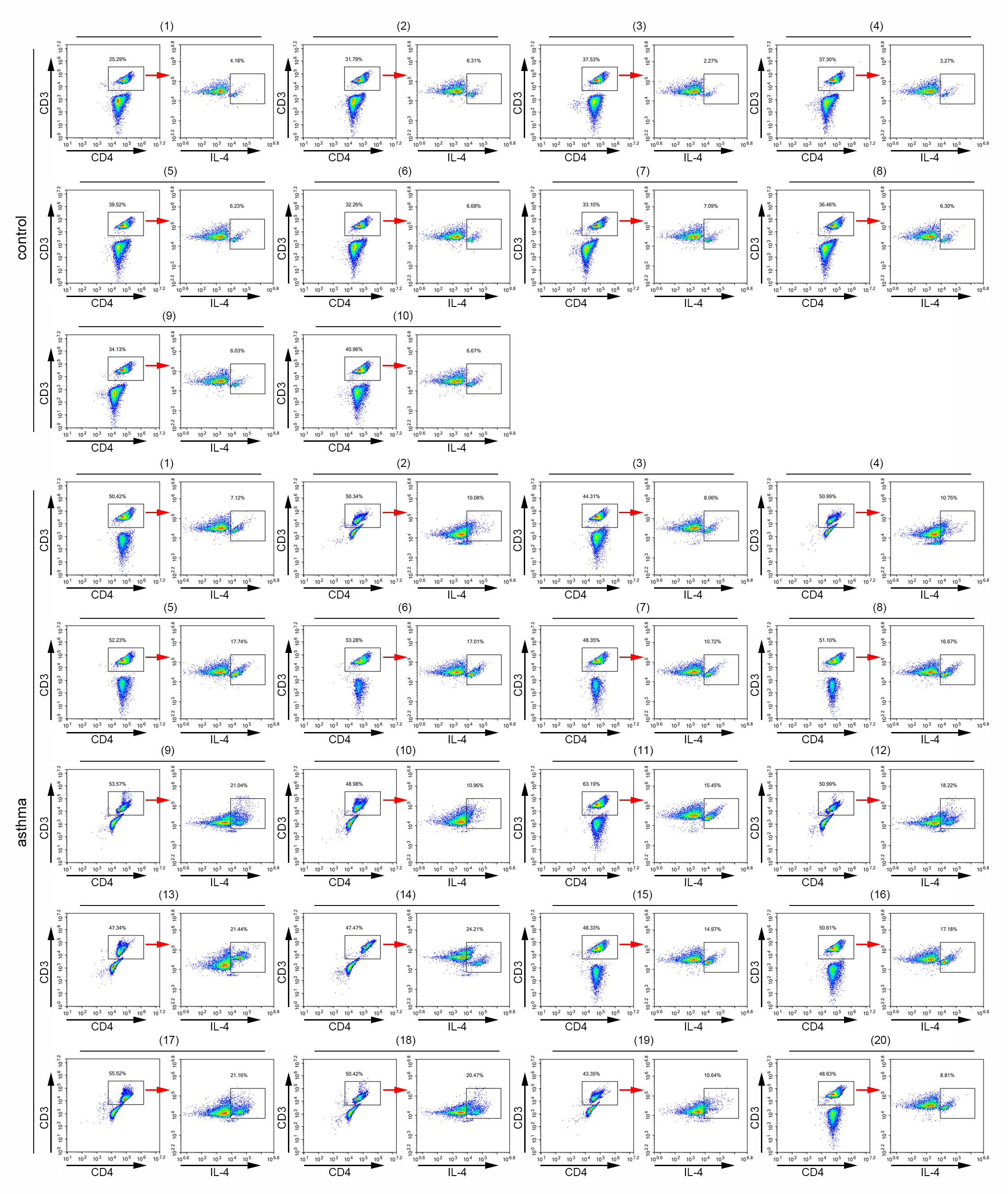

Supplement: Supplementary file 2 — Supplementary Material 2 [file 10020_2024_872_MOESM2_ESM.png]
